# Comparison of sequential therapy and amoxicillin/tetracycline containing bismuth quadruple therapy for the first-line eradication of *Helicobacter pylori*: a prospective, multi-center, randomized clinical trial

**DOI:** 10.1186/s12876-016-0490-8

**Published:** 2016-07-26

**Authors:** Ju Yup Lee, Nayoung Kim, Kyung Sik Park, Hyun Jin Kim, Seon Mee Park, Gwang Ho Baik, Ki-Nam Shim, Jung Hwan Oh, Suck Chei Choi, Sung Eun Kim, Won Hee Kim, Seon-Young Park, Gwang Ha Kim, Bong Eun Lee, Yunju Jo, Su Jin Hong

**Affiliations:** 1 Department of Internal Medicine, Seoul National University Bundang Hospital, Seongnam, South Korea; 2 Department of Internal Medicine, Keimyung University School of Medicine, Daegu, South Korea; 3 Department of Internal Medicine and Institute of Health Science, Gyeongsang National University School of Medicine, Jinju, Gyeongsangnam-do South Korea; 4 Department of Internal Medicine, College of Medicine, Chungbuk National University, Cheongju, South Korea; 5 Department of Internal Medicine, Hallym University College of Medicine, Chuncheon Sacred Heart Hospital, Chuncheon, South Korea; 6 Department of Internal Medicine, Ewha Womans University School of Medicine, Seoul, South Korea; 7 Departments of Internal Medicine, College of Medicine, The Catholic University of Korea, Seoul, Republic of Korea; 8 Department of Internal Medicine, Wonkwang University School of Medicine, Iksan, South Korea; 9 Department of Internal Medicine, Kosin University College of Medicine, Busan, South Korea; 10 Digestive Disease Center, CHA Bundang Medical Center, CHA University, Seongnam, South Korea; 11 Department of Internal Medicine, Chonnam National University Medical School, Gwangju, South Korea; 12 Department of Internal Medicine, Pusan National University School of Medicine and Biomedical Research Institute, Pusan National University Hospital, Busan, South Korea; 13 Department of Internal Medicine, Eulji General Hospital, Eulji University School of Medicine, Seoul, South Korea; 14 Department of Internal Medicine and Research Institute, Soonchunhyang University College of Medicine, Bucheon, South Korea

**Keywords:** *Helicobacter pylori*, Eradication, Amoxicillin, Tetracycline, Bismuth, Quadruple

## Abstract

**Background:**

The <80 % *Helicobacter pylori* eradication rate with sequential therapy is unsatisfactory. Modified bismuth quadruple therapy, replacing metronidazole with amoxicillin, could be promising because *H. pylori* resistance to tetracycline or to amoxicillin is relatively low. A 14-day modified bismuth quadruple protocol as first-line *H. pylori* treatment was compared with 10-day sequential therapy.

**Methods:**

In total, 390 *H. pylori*-infected subjects participated in the randomized clinical trial: 10-day sequential therapy (40 mg pantoprazole plus 1 g amoxicillin twice a day for 5 days, then 40 mg pantoprazole and 500 mg clarithromycin twice a day and 500 mg metronidazole three times a day for 5 days) or 14-day modified bismuth quadruple therapy (40 mg pantoprazole, 600 mg bismuth subcitrate, 1 g tetracycline, and 1 g amoxicillin, twice a day). ^13^C-urea breath test, rapid urease testing, or histology was performed to check for eradication.

**Results:**

Intention-to-treat (ITT) eradication rates of 10-day sequential and 14-day quadruple therapy were 74.6 % and 68.7 %, respectively, and the per-protocol (PP) rates were 84.2 and 76.5 %, respectively. The eradication rate was higher in the sequential therapy group, but neither the ITT nor the PP analyses had a significant difference (*P* = 0.240 and *P* = 0.099, respectively). However, the adverse events were significantly lower in the modified bismuth quadruple therapy group than the sequential therapy group (36.9 vs. 47.7 %, *P* = 0.040).

**Conclusions:**

Ten-day sequential therapy appears to be more effective despite frequent adverse events. However, both 10-day SQT and 14-day PBAT did not reach the excellent eradication rates that exceed 90 %. Additional trials are needed to identify a more satisfactory first-line eradication therapy.

**Trial registration:**

ClinicalTrials.gov (NCT02159976); Registration date: 2014-06-03, CRIS (KCT0001176); Registration date: 2014-07-23.

**Electronic supplementary material:**

The online version of this article (doi:10.1186/s12876-016-0490-8) contains supplementary material, which is available to authorized users.

## Background

*Helicobacter pylori* is a major cause of gastric diseases such as chronic gastritis, gastroduodenal ulcers, and gastric cancer, and it is well known that the eradication of *H. pylori* is important in preventing and treating these gastric diseases [1, 2]. Recently, the Kyoto Global Consensus Meeting, held in Japan in early February of 2014, presented radical changes in the diagnosis and treatment of *H. pylori* infection, such as recommending eradication treatment for patients with dyspepsia [3]. In addition, eradication therapy was recommended for all *H. pylori*-positive individuals for the purpose of preventing *H. pylori-*related diseases [3]. However, the eradication rates of first-line triple therapy, which consists of a proton pump inhibitor (PPI) and two antibiotics (clarithromycin and amoxicillin), have been continuously decreasing [4]. Only ~18 % of previous studies have reported exceeding 85 % eradication on an intention-to-treat (ITT) analysis, with ~60 % falling short of 80 % [5]. The reason for the decrease in the efficacy of PPI-based triple therapy is mainly due to the increase in *H. pylori* resistance to clarithromycin. As such, in Western countries, standard triple therapy is currently considered a “legacy therapy” [5]. In fact, recent European guidelines recommended triple therapy as the first-line treatment only when the prevalence of clarithromycin resistance is under 20 % [6].

To overcome this unsatisfactory eradication rate, sequential therapy (SQT) is currently recommended as an alternative first-line treatment for *H. pylori* infection. Many European randomized clinical trials (RCTs) and meta-analyses have shown the superiority of SQT over standard PPI-based triple therapy [7–9]. In a Korean meta-analysis based on six RCTs, the eradication rate of SQT was 79.4 % in ITT analysis and 86.4 % in PP analysis; this meta-analysis also proved that SQT is superior to standard PPI-based triple therapy (relative risk [RR] 1.761, 95 % confidence interval [CI]; 1.403–2.209) [10]. However, the eradication rate of SQT in Korea, mostly under 80 % (ITT: 79.4 %, 95 % CI: 76.3–82.2) [10], is not satisfactory and is about 10 % lower than reported in early European studies [10, 11]. This suboptimal eradication rate could be explained by high rates of antibiotic resistance, especially to clarithromycin, metronidazole, or both [12]. Previous studies showed that the rate of resistance to clarithromycin was >20 %, that to metronidazole was >30 %, and that to both was >10 % [13, 14].

The classical bismuth quadruple therapy, which consists of a PPI, bismuth, tetracycline, and metronidazole, is frequently used as a first- or second-line regimen [6, 15–17]. However, it is known that more than 30 % of patients stop taking their medicine due to the complicated regimen and the high rate of adverse events [18]. In view of antibiotic resistance, a modified bismuth quadruple therapy (PBAT), in which metronidazole is replaced with amoxicillin, is very attractive because many studies have reported that *H. pylori* resistance to amoxicillin and tetracycline is low [19] and furthermore, amoxicillin is easier to take in comparison to metronidazole. Several studies have suggested good eradication rates with this amoxicillin and tetracycline combined quadruple regimen [20–23]; however, the reported efficacies are conflicting. From this background the aim of this study was to evaluate the efficacy of 14-day PBAT as a first-line treatment and compare 10-day SQT to 14-day PBAT in order to establish a more effective first-line regimen for *H. pylori* in terms of eradication rate and adverse effects.

## Methods

### Study design and participants

A prospective, multi-center, randomized, open-label, parallel design clinical trial was conducted between July 2014 and May 2015 in 14 tertiary hospitals from different regions in Korea. Inclusion criteria were adult Korean men and women (aged ≥ 18 years) who were diagnosed as *H. pylori*-positive by any of the following three methods: 1) a positive rapid urease test (CLOtest [Delta West, Bentley, Australia]), 2) histologic evidence of *H. pylori* by modified Giemsa staining, or a 3) positive ^13^C-urea breath test. Exclusion criteria were 1) age under 18 years; 2) previous eradication therapy for *H. pylori*; 3) drugs that could influence the study results such as a PPI, H_2_ blocker, mucosal protective agent, or antibiotics within the prior 4 weeks; 3) previous gastric surgery; 4) advanced gastric cancer or other malignancy; 5) abnormal liver function or liver cirrhosis; 6) abnormal renal function or chronic kidney disease; 7) other severe concurrent diseases; 8) previous contraindications or allergic reactions to the study drugs; 9) genetic disorders such as galactose intolerance, the Lapp lactase deficiency, or glucose-galactose malabsorption; 10) mental disorders or alcohol or drug addiction; 11) pregnancy or lactation or refusal to use an appropriate method of contraception throughout the course of the study; 12) any condition that might affect the evaluation of the clinical results in the judgment of the principal investigator or sub-investigator; or 13) any specific contraindication to the study drugs.

The study protocol was approved by the Korean Food and Drug Administration (KFDA No. 30157) and by the Institutional Review Board and Ethics Committees of all participating hospitals (Additional file 1). The study was performed according to Good Clinical Practices (GCP) and the Declaration of Helsinki, and written informed consent was obtained from all patients before enrollment. In addition, this study protocol has been registered at ClinicalTrials.gov (NCT02159976) and Clinical Research Information Service (CRIS) (KCT0001176).

### Randomization and treatment allocation

An independent statistician at Seoul National University Bundang Hospital (SNUBH) prepared the randomization. The subjects were randomized in a 1:1 ratio by a block randomization method to receive either 10-day SQT or 14-day PBAT. The 10-day SQT consisted of 40 mg of pantoprazole plus 1 g of amoxicillin twice a day for the initial 5 days, followed by 40 mg of pantoprazole and 500 mg of clarithromycin twice a day and 500 mg of metronidazole three times a day for the subsequent 5 days. The 14-day PBAT consisted of 40 mg of pantoprazole, 600 mg of bismuth subcitrate, 1 g of tetracycline, and 1 g of amoxicillin, twice a day. Four weeks after completing the therapy, successful *H. pylori* eradication was defined by a negative ^13^C-urea breath test or an invasive test when endoscopic follow-up was needed in cases of benign gastric ulcer. The drug compliance and adverse events were evaluated by a physician via direct questioning. Compliance was considered to be satisfactory when drug intake exceeded 85 %.

### Assessment of *H. pylori* infection

#### Invasive *H. pylori* test (Giemsa histology and CLOtest)

To determine the presence of current *H. pylori* infection, four biopsy specimens (one each from the greater curvature and lesser curvature of the antrum and body) were taken from the gastric mucosa at each endoscopic examination and were fixed in formalin to be used for the evaluation of *H. pylori* infection by Giemsa staining [24]. Two specimens from the lesser curvature of the antrum and body were used for the rapid urease test (CLOtest) [24].

#### ^13^C-urea breath test

Subjects were fasted for 4 h prior to testing and a pre-dose breath sample was obtained; 100 mg of ^13^C-urea powder (UBiTkit; Otsuka Pharmaceutical Co. Ltd., Tokyo, Japan) dissolved in 100 mL of water was then administered orally, and a second breath sample was collected 20 min later. The cutoff value was 2.5 %. The collected samples were analyzed using an isotope ratio mass spectrometer (UBiT-IR300; Otsuka Pharmaceutical Co., Ltd.).

### Trial outcomes

The primary outcome was comparing the percentage of participants with successful *H. pylori* eradication in the 10-day SQT and 14-day PBAT groups 4–6 weeks after completion of eradication therapy. The secondary outcome was to compare the percentage of patients whose drug compliance was greater than 85 % and the percentage of adverse events in the 10-day SQT and 14-day PBAT groups.

### Sample size and statistical analysis

Because the eradication rate of the 10-day SQT was found to be 82.0 % in a previous Korean report [10] the eradication rate of the 14-day PBAT was assumed to be similar to that of the 10-day SQT if the rate difference between the two regimens was less than 10 %. With a power of 80 % at a two-sided type 1 error rate of 5 %, 195 subjects were needed for each treatment arm to allow for 10 % loss to follow-up. The *H. pylori* eradication rate was determined by both an ITT and a per-protocol (PP) analysis. All subjects who received treatment were included in the ITT analysis. For the PP analysis, subjects who were lost to follow-up, had taken less than 85 % of the prescribed drugs, or had dropped out due to severe adverse events were excluded.

Parametric continuous variables were compared using the Student’s *t*-test and are presented as mean ± standard deviation (SD). Categorical variables were analyzed using Pearson’s chi square test or Fisher’s exact test and were presented as numbers (percentages). Univariate and multivariate logistic regression were used for analysis of influencing factors, which were expressed as the odds ratios (OR) and 95 % confidence intervals (CI). A two-sided *P* value of less than 0.05 was considered statistically significant. All statistical analyses were performed using SPSS (version 20.0; SPSS Inc., Chicago, IL, US).

## Results

### Subjects

**Fig. 1:**
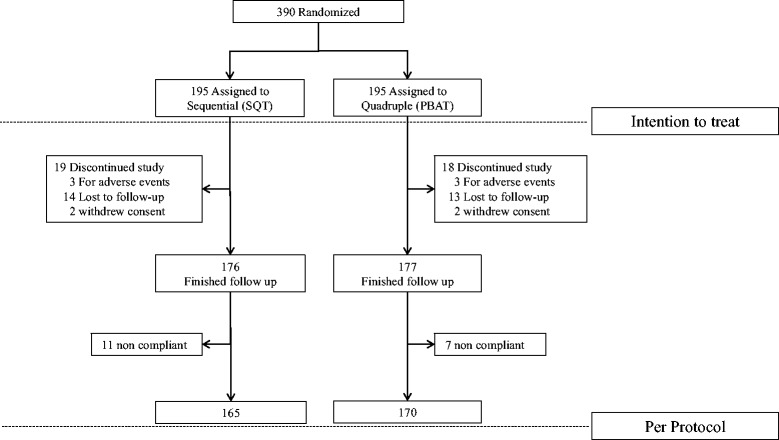
Flowchart of participants

A total of 390 *H. pylori*-infected treatment-naïve subjects were randomly assigned to the 10-day SQT group (*n* = 195) or the 14-day PBAT group (*n* = 195) (Fig. 1). Nineteen subjects in the SQT group and 18 subjects in the PBAT group did not complete the study due to adverse events, loss of follow-up, or withdrawal of consent. Therefore, 176 subjects in the SQT group and 175 subjects in the PBAT group completed the follow-up. After exclusion of 11 and 7 non-compliant subjects who consumed <85 % of the prescribed medications, 165 and 170 subjects in the SQT and PBAT groups, respectively, became the objects of the PP analysis (Fig. 1). There were no significant differences in the baseline characteristics or endoscopic findings between the two groups (Table 1).

**Table 1:** Baseline characteristics of the subjects

	SQT (*n* = 195)	PBAT (*n* = 195)	*p-*value
Gender, n (%)			0.184
Male	103 (52.8)	117 (60.0)	
Female	92 (47.2)	78 (40.0)	
Age (mean ± SD), year	53.1 ± 12.6	53.6 ± 13.2	0.697
BMI, kg/m^2^	23.5 ± 3.1	23.9 ± 3.4	0.328
Smoking, n (%)	38 (19.5)	36 (18.5)	0.897
Alcohol, n (%)	75 (38.5)	72 (36.9)	0.835
Endoscopic finding, n (%)			
Normal	10 (5.1)	11 (5.6)	1.000
Atrophic gastritis with or without intestinal metaplasia	43 (22.1)	31 (15.9)	0.155
Other gastritis	12 (6.2)	16 (8.2)	0.556
Gastric ulcer	47 (24.1)	43 (22.1)	0.718
Duodenal ulcer	15 (7.7)	20 (10.3)	0.479
Gastric ulcer + Duodenal ulcer	4 (2.1)	7 (3.6)	0.541
EMR, ESD for dysplasia or EGC	16 (8.2)	18 (9.2)	0.858
MALToma	1 (0.5)	0 (0.0)	1.000
Reflux esophagitis	1 (0.5)	3 (1.5)	0.615
Others	10 (5.1)	7 (3.6)	0.620

*SQT* sequential therapy, *PBAT* quadruple therapy consist of pantoprazole, bismuth, amoxicillin, and tetracycline, *BMI* body mass index, *EMR* endoscopic mucosal resection, *ESD* endoscopic submucosal dissection, *EGC* early gastric cancer, *MALToma* mucosal associated lymphoid tissue lymphoma

### Eradication rates

**Fig. 2:**
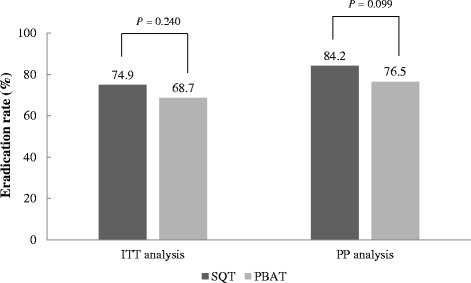
*H. pylori* eradication rate of 10-day SQT and 14-day PBAT according to the ITT and PP analyses. SQT, sequential therapy; PBAT, quadruple therapy consists of pantoprazole, bismuth, amoxicillin, and tetracycline; ITT, intention-to-treat; PP, per-protocol

**Fig. 3:**
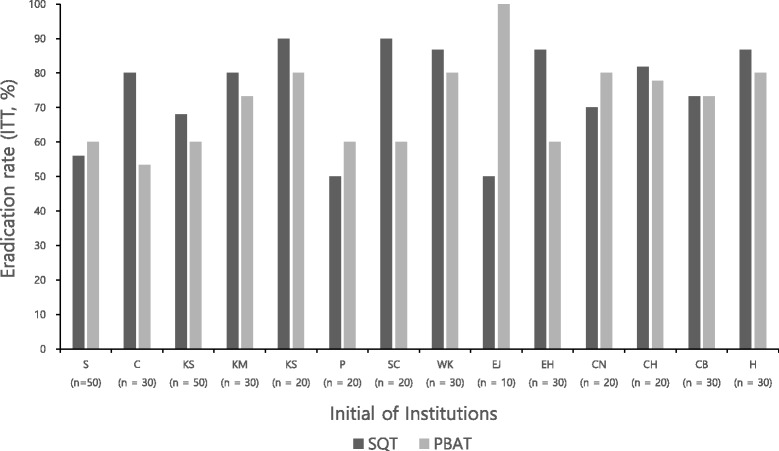
*H. pylori* eradication rate of 10-day SQT and 14-day PBAT according to each institution. SQT, sequential therapy; PBAT, quadruple therapy consists of pantoprazole, bismuth, amoxicillin, and tetracycline; ITT, intention-to-treat

ITT eradication rates of the 10-day SQT and 14-day PBAT groups were 74.6 % (146/195) and 68.7 % (134/195) and the PP eradication rates were 84.2 % (139/165) and 76.5 % (130/170), respectively. The eradication rate was higher in the SQT than the PBAT group, but there was no statistical significance on either ITT or PP analysis (*P* = 0.240 and *P* = 0.099, respectively) (Fig. 2). There also was no statistical significance in the ITT analysis conducted independently at each institution (Fig. 3).

### Compliance and adverse events

The complete follow-up rate was 90.3 % (176/195) in the 10-day SQT and 90.8 % in the 14-day PBAT group. The numbers of subjects who took more than 85 % of the prescribed medicine were 93.8 % (165/176) in the 10-day SQT group and 96.1 % (170/177) in the 14-day PBAT group. There was also no significant difference between the two groups (*P* = 0.460). Ninety-three patients (47.7 %) who took the sequential regimen and 72 patients (36.9 %) who took the quadruple regimen experienced at least one adverse event. The adverse event rate was significantly lower in the PBAT group than in the SQT group (*P* = 0.040). In the 10-day SQT group, the most frequent adverse events were taste distortion (25.8 %) and abdominal bloating (22.6 %). In the 14-day PBAT group, the most frequent adverse events were epigastric discomfort (23.6 %) and diarrhea (16.7 %). Taste distortion was more frequently reported in the 10-day SQT group (*P* < 0.001) and diarrhea was more frequently reported in the 14-day PBAT group (*P* = 0.014) (Table 2).

**Table 2:** Adverse events of the subjects

	SQT (*n* = 195)	PBAT (*n* = 195)	*p-*value
	n (%)	n (%)	
Bloating	21 (22.6)	11 (15.3)	0.097
Epigastric soreness	15 (16.1)	17 (23.6)	0.854
Anorexia	2 (2.2)	1 (1.4)	1.000
Taste distortions	24 (25.8)	4 (5.6)	**<0.001**
Nausea	14 (15.1)	6 (8.3)	0.108
Vomiting	0	2 (2.8)	0.478
Abdominal pain	4 (4.3)	4 (5.6)	0.721
Headache	1 (1.1)	2 (2.8)	1.000
Dyspepsia	4 (4.3)	4 (5.6)	0.721
Diarrhea	2 (2.2)	12 (16.7)	**0.014**
Constipation	2 (2.2)	3 (4.2)	1.000
Reflux	1 (1.1)	1 (1.4)	0.478
Rash and itching	2 (2.2)	5 (2.8)	0.446
Dizziness	1 (1.1)	0	1.000
Stool color change	0	3 (4.2)	0.246
Total	93 (47.7)	72 (36.9)	**0.040**

Bold style, means statistical significance

*SQT* sequential therapy, *PBAT* quadruple therapy consist of pantoprazole, bismuth, amoxicillin, and tetracycline

### Factors associated with eradication failure in PBAT

On univariate analysis, male sex (OR, 0.38; 95 % CI, 0.31–1.30) and BMI ≥ 25 kg/m^2^ (OR, 2.28; 95 % CI, 1.12–4.63) were associated with treatment failure in the 14-day PBAT group. The multivariate analysis confirmed that both male sex (OR, 0.27; 95 % CI, 0.34–1.51) and BMI (OR, 2.16; 95 % CI, 1.05–4.43) were associated with treatment failure in the 14-day PBAT group (Table 3).

**Table 3:** Factors associated with eradication failure in amoxicillin- and tetracycline containing quadruple therapy

Factors	Crude	Univariated	Adjusted	Multivariated
	OR (95 % CI)	*p-*value^a^	OR (95 % CI)^b^	*p-*value^c^
Age ≥ 50 (y)	1.12 (0.54–2.32)	0.763		
Female	0.38 (0.31–1.30)	<0.0001	0.27 (0.34–1.51)	<0.0001
Cigarette smoking	1.35 (0.57–3.20)	0.499		
Alcohol drinking	1.42 (0.70–2.88)	0.325		
BMI ≥ 25 (kg/m^2^)	2.28 (1.12–4.63)	0.023	2.16 (1.05–4.43)	0.037

*OR* odds ratio, *CI* confidence intervals, *BMI* body mass index

^a^Univariate logistic regression

^b^Adjusted for gender and BMI

^c^Multivariate logistic regression

## Discussion

Antibiotic resistance is one of the main causes of treatment failure in *H. pylori* eradication. The rates of clarithromycin resistance increased rapidly from 23.2 to 37.3 % from 2003 to 2012 and the metronidazole resistance rate was 35.8 % between 2009 and 2012 in Korea [4]. In contrast, the rates of resistance to amoxicillin and tetracycline are relatively low, at 17.2 and 10.8 %, respectively [4]. The average resistance rate to amoxicillin in Europe is reported to be lower than 2 %, and the resistance rate to tetracycline is reported to be below 5 % in most countries [25–27].

We hypothesized that a quadruple regimen containing amoxicillin and tetracycline would demonstrate a superior eradication rate and could be a suitable substitute for triple or sequential regimens in the first-line treatment of *H. pylori* infection. The ITT eradication rate of the 14-day PBAT was 68.7 % and that of 10-day SQT was 74.6 %. There was no statistically significance difference between these two treatment groups (*P* = 0.240), suggesting that 14-day PBAT was not inferior to 10-day SQT and therefore 14-day PBAT could be another treatment option.

However, some controversies in the results and large heterogeneity in the duration or the first- or second-line regimen exists in previous studies of amoxicillin- and tetracycline-containing quadruple regimens. For instance, in a study of the 14-day quadruple regimen, the eradication rate was 33.3 % in amoxicillin-susceptible *H. pylori*-infected patients [28]. In contrast, a Chinese study showed that the eradication rate of 14-day LBAT (lansoprazole, bismuth, amoxicillin, and tetracycline) was 83.8 % (95 % CI: 76.8–90.9 %) [20]. Furthermore, an RCT performed in Turkey reported the eradication rate of 14-day EBAT (esomeprazole, bismuth, amoxicillin, and tetracycline) as 79.0 % (95 % CI: 71–87 %) [21]. Chi et al. [23] reported that quadruple therapy containing amoxicillin and tetracycline is an effective regimen to rescue patients after a failed triple therapy by overcoming the antimicrobial resistance of *H. pylori* with an eradication rate of 78 % in ITT and 89 % in PP.

Since *H. pylori* resistance to amoxicillin and tetracycline is uncommon, the explanation for the heterogeneous results could be that both amoxicillin and tetracycline are weak against *H. pylori* or possibly there is an antagonistic effect. However, any possible antagonistic effect between these two antibiotics is unclear and it is difficult to elucidate because data on the gastric bioavailability of tetracycline is lacking [29]. Furthermore, several studies showed a good eradication rate (78.0–83.8 %), which was not inferior to other regimens, and this positive tendency was also proven in a recent meta-analysis. Published in 2015, the meta-analysis, which included 9 RCTs, showed that the total eradication rate of a quadruple regimen containing amoxicillin and tetracycline was 78.1 % in ITT and 84.5 % in PP, which was not inferior to other quadruple regimens (pooled odds ratio [OR]: 0.9, 95 % CI: 0.42–1.78) [30]. In subgroup analysis, the eradication rates of 7-day, 10-day, and 14-day amoxicillin/tetracycline quadruple regimens were 67.4, 84.6, and 82.3 %, respectively, and the pooled OR of an amoxicillin/tetracycline quadruple regimen used as first-line therapy was 2.34 (95 % CI: 0.74–7.42) [30].

In our study, we used different doses and intervals of PBAT compared with those of previous studies to maximize subject compliance. In previous studies, bismuth was administered at 300 mg and tetracycline at 500 mg, both four times a day [21, 31]. The mixture of twice a day and four times a day can influence drug compliance. A recently developed three-in-one capsule containing bismuth, tetracycline, and metronidazole improved the eradication rate of bismuth quadruple therapy over 90 % by reducing the number of medicines and therefore improving patient compliance [32]. In the present clinical trial bismuth 600 mg twice a day and tetracycline 1000 mg twice a day were chosen to increase compliance.

Tetracycline is a time-dependent (half-life 8–10 h) antibiotic agent and has a long post-antibiotic effect (PAE) [33]. Even though a high blood concentration of tetracycline does not increase its sterilizing power, it can inhibit bacterial regrowth for a longer period of time [34, 35]. From these pharmacokinetic considerations, a twice-a-day regimen is also appropriate and is likely to promote patient compliance. The drug compliance in the present study was 96.1 % in the PBAT group, which is comparable to that in the SQT group (93.8 %). In addition, this rate is higher than the 88.6 % [20] and 92.0 % [31] reported by other studies.

This clinical trial had some limitations. First, *H. pylori* culture was not routinely performed and we could not evaluate the eradication rate of amoxicillin- and/or tetracycline-susceptible *H. pylori* strains. However, one center (SNUBH) performed *H. pylori* cultures and antibiotic susceptibility testing by the agar dilution method in a small number of patients. The first-line or second-line eradication rate of PBAT was 57.1 % (8/14) in amoxicillin- and tetracycline-susceptible subjects (data not shown). More antibiotic susceptibility data are needed in a future study to confirm the eradication effect of amoxicillin and tetracycline-containing regimens both in vitro and in vivo. Second, the limited accuracy of ^13^C-UBT at 4–6 weeks after the completion of a bismuth-based treatment that makes possible, at least in part, a false negative diagnosis of *H. pylori* eradication leading to an overestimation of success rates with the bismuth-based regimen (PBAT). However, ^13^C-UBT is a convenient non-invasive method for *H. pylori* diagnosis and popularly used for eradication success. It is very difficult to perform invasive *H. pylori* test such as histology or CLOtest via upper endoscopy to all patients in real clinical setting who did not need follow-up endoscopy and medical cost is also a problem. Third, we cannot elucidate the reason why PBAT showed a lower eradication rate than expected. However, there is a fairly large eradication rate gap between positive and negative studies. Further study is needed in the future.

## Conclusions

In conclusion, this prospective, multi-center, randomized, open-label, parallel design clinical trial demonstrated that SQT for 10 days appears more effective than PBAT in spite of frequent adverse events. However, both 10-day SQT and 14-day PBAT did not achieve excellent eradication rates (>90 %). Thus, additional trials are necessary to identify a more satisfactory first-line eradication therapy for *H. pylori.*

## Abbreviations

EGC, early gastric cancer; EMR, endoscopic mucosal resection; ESD, endoscopic submucosal dissection; *H. pylori*, *Helicobacter pylori*; MALToma, mucosal associated lymphoid tissue lymphoma; PBAT, quadruple therapy consist of pantoprazole, bismuth, amoxicillin, and tetracycline; SQT, sequential therapy

## Additional file

Additional file 1: Lists of the IRBs. (DOCX 15 kb)
