# Supplementary material for: Comparison of sequential therapy and amoxicillin/tetracycline containing bismuth quadruple therapy for the first-line eradication of Helicobacter pylori: a prospective, multi-center, randomized clinical trial
Source: BMC Gastroenterol. 2016 Jul 26;16:79. doi: 10.1186/s12876-016-0490-8 (PMC4962407; doi:10.1186/s12876-016-0490-8)
Supplement: Additional file 1: — Lists of the IRBs. (DOCX 15 kb) [file 12876_2016_490_MOESM1_ESM.docx]

**Lists of the institutional review boards**

1. Seoul National University Bundang Hospital Institutional Review Board
2. Keimyung University Dongsan Medical Center Institutional Review Board
3. Gyeongsang National University Hospital Institutional Review Board
4. Chungbuk National University Hospital Institutional Review Board
5. Hallym University Chuncheon Sacred Heart Hospital Institutional Review Board
6. Ewha Womans University Mokdong Hospital Institutional Review Board
7. The Catholic University of Korea St. Paul’s Hospital Institutional Review Board
8. Wonkwang University Hospital Institutional Review Board
9. Kosin University Hospital Institutional Review Board
10. CHA University Hospital Institutional Review Board
11. Chonnam National University Hospital Institutional Review Board
12. Pusan National University Hospital Institutional Review Board
13. Eulji General Hospital Institutional Review Board
14. Soonchunhyang University Buchen Hospital Institutional Review Board
